# Clinical significance of glomerular IgM deposit in IgA nephropathy: a 5-year follow-up study

**DOI:** 10.1080/0886022X.2024.2386146

**Published:** 2024-08-01

**Authors:** Ziyuan Huang, Jiayan Xu, Jianwei Ma, Chenyi Yuan, Qin Su, Yudong Chu, Jiancheng Huang, Xueyan Bian

**Affiliations:** a Department of Nephrology, The First Affiliated Hospital of Ningbo University, Ningbo, Zhejiang, PR China; b Institute of Chronic Kidney Disease, Medical University, Ningbo, Zhejiang, PR China

**Keywords:** Prognosis, clinicopathology, IgA nephropathy, glomerular IgM deposit

## Abstract

The significance of glomerular IgM deposit intensity in IgA Nephropathy (IgAN) remained ambiguous and requires further research. Patients with biopsy-proven IgAN in our hospital from January 2018 to May 2023 were recruited into this retrospective single-center study. Patients who presented with positive IgM deposit were included in IgM + cohort while patients with negative IgM deposit were included in IgM– cohort. Of the IgM+, patients whose IF intensity of IgM deposits exceeded 1+ formed IgM-H cohort while patients whose IF intensity of IgM deposits was equal to 1+ consisted IgM-L cohort. Pairwise comparisons were performed among these cohorts to determine clinical disparities, following the propensity score matching process. Among 982 IgAN patients, 539 patients presented with positive IgM deposit. The Kaplan–Meier analysis showed that the IgM deposit did not contribute adversely to the outcomes (eGFR decreased from the baseline ≥ 50% continuously or reached end-stage renal disease). However, the Cox regression analysis showed that increased intensity of IgM deposit was an independent risk factor (*p* = 0.03) in IgM+. The IgM-H exhibited more pronounced segmental glomerulosclerosis (*p* = 0.02) than the IgM-L, which may also be associated more directly with higher urine protein levels (*p* = 0.02). Moreover, our generalized linear mixed model demonstrated a remarkably higher urine albumin/creatinine ratio (*p* < 0.01) and serum creatinine (*p* = 0.04) levels as well as lower serum albumin (*p* < 0.01) level in IgM-H persistently during the 5-year follow-up. This study concluded that increased intensity of glomerular IgM deposits may contribute adversely to clinicopathologic presentation and outcome in those IgM + patients.

## Introduction

IgA nephropathy (IgAN) is considered the most common primary glomerulonephritis worldwide and 25–30% of patients with IgAN will progress to end-stage renal disease (ESRD) [1,2]. However, its various pathological manifestations complicate the prediction of patient prognosis and the efficacy of therapy, requiring further studies. IgAN has been proposed to develop through the overproduction of galactose-deficient IgA1 (Gd-IgA1) and formation of polymeric IgA1 immune complexes, which deposit in the glomerular mesangium and lead to kidney inflammation. The significance of the immuno-staining pattern and intensity was one of the worthy topics [3]. While glomerular IgM deposit was observed in approximately 25–80% of IgAN patients [4–7], its potential impact on disease progression was less explored and remained unclear. Previous studies have yielded conflicting results. Liu et al. [8] revealed that frequency of IgM deposit in renal glomeruli increased remarkably at the repeated renal biopsies during the course of disease. They stated that the glomerular IgM deposits in IgAN was a secondary phenomenon which may lead to poorer outcomes. Previous studies [9,10] also showed that the higher prevalence of IgM deposition was a poor renal survival indicator in patients with nephrotic-range proteinuria. Stefan et al. [7] considered the glomerular IgM deposit as a key risk factor for ESRD in IgAN, particularly in advanced stages of the disease. Heybeli et al. [4] proposed mesangial IgM deposition was more frequently associated with resistant disease in IgAN patients. However, Xiong et al. [11] found that glomerular IgM deposits in pediatric IgAN cases didn’t contribute adversely to the outcome. Moriyama et al. proposed [9] that although glomerular IgM deposits were associated with certain chronic glomerular lesions, it did not affect renal survival in the short term. These findings from various researches are interesting but confusing. Thus, our study aimed to explore further the clinical significance of glomerular IgM deposits and its intensity as well as their influence on prognosis. Patients enrolled in this research were categorized into several cohorts according to the intensity of glomerular IgM deposits, and pairwise comparisons were performed following the propensity score matching (PSM) process [12]. Also, the larger sample size and long follow-up time of this research facilitate systematic reviews. The findings from this study can provide valuable insights for the clinical diagnosis and treatment of IgAN.

## Methods

### Patient profiles

A retrospective review of data from 1101 cases of biopsy-proven primary IgAN at our hospital January 2018 and May 2023 was conducted. Patients with other primary or secondary renal diseases have been ruled out in this study according to their renal biopsies. The selection criteria were as follows: (1) a follow-up duration of >6 months after the renal biopsy; (2) renal biopsy ≥8 glomeruli and (3) availability of complete clinicopathological data required for this study. Patients who met the selection criteria were finally recruited into this study. Among them, patients with the presence of glomerular IgM deposits formed the IgM + cohort while the patients with negative IgM deposits consisted the IgM– cohort. The glomerular IgM deposits in this research refer to the deposits on the mesangial cells. Of the patients in the IgM + cohort, patients whose IF intensity of IgM deposits exceeded 1+ were included in the IgM-H cohort. The remaining patients, whose IF intensity of IgM deposits was equal to 1+, were included in the IgM-L cohort. Pairwise comparisons were performed among these cohorts to determine their prognostic and clinicopathological disparities, following the propensity score matching (PSM) process. This is a single-center retrospective cohort study, which was approved by the Ethics Committee of the First Affiliated Hospital of Ningbo University. All data were collected from the information system of the hospital, and patient-identifiable information was anonymized.

### Data collection

Data concerning age, sex, weight, height, blood pressure, albumin, serum creatinine, uric acid, triglyceride, total cholesterol, LDL-cholesterol, HDL-cholesterol, platelet count, neutrophil count, lymphocyte count, serum immunoglobulin (IgA, IgG and IgM), serum complement (C3 and C4), 24-h urinary protein (UP), and urinary albumin/creatinine ratio (UACR) were collected and regarded as baseline measurements. Throughout the follow-up phase, additional data on use of medication (renin-angiotensin-aldosterone system blockade, steroids and immunosuppressants), serum creatinine, albumin, urinary albumin/creatinine ratio levels were recorded at every visit.

All kidney biopsy specimens were examined using light microscopy (LM) and IF. The pathologic diagnosis according to LM findings was assesed by 2 experienced pathologists following the Oxford classification criteria [1]. The classification contains mesangial hypercellularity (M0/1), endocapillary hypercellularity (E0/1), tubular atrophy and interstitial fibrosis (T0/1/2), segmental glomerulosclerosis (S0/1), and crescent (C0/1). For IF, the sections were dripped with human IgM monoclonal antibody (1:100; cat nos. ab212201; Abcam, Cambridge, MA, USA). The sections were then incubated with Alexa Fluor^®^ 488 goat anti-rabbit secondary antibody (1:200; Cat nos. ab150077; Abcam, Cambridge, MA, USA), and imaged using fluorescence microscopes. Based on the IF results, the staining intensities of immunoglobulin and complement deposits were assessed. The staining intensities were classified as follows: −, trace, +, ++, +++ and ++++ [13] (Figure S1).

### Definitions

The systemic immune-inflammation index (SII) was derived from platelet count × neutrophil count/lymphocyte count [14]. During the follow-up period, the average urinary albumin/creatinine ratio and SII was calculated for each 6-month block (the part <6 months was regarded as one block) [15]. The average of this calculated value was considered as the time-average albumin/creatinine ratio (TA UACR) and the time-average systemic immune-inflammation index (TA SII), respectively. The eGFR was calculated using the CKD-EPI Creatinine Equation (2021) [16]. The composite endpoint was characterized by a sustained decline in eGFR from a baseline ≥ 50% or reaching ESRD.

### Statistical analysis

The numerical variables were presented as the means (SD) or medians [interquartile range], and their comparisons were expressed using variance analysis or the Kruskal-Wallis rank test, according to the distribution of the data. Categorical data were expressed as counts (%) and compared using Pearson’s chi-squared test. The correction of multiple comparisons was performed using the Benjamini-Hochberg method [17]. Moreover, violin plots were performed to present the distribution of clinical indicator levels across different subgroups. Moreover, the linear data was categorized into three levels (low, medium, and high) according to the tertiles and were sent for correlation analysis together with other ranked data. The correlation coefficient (Kendall’s method for ranked data) was calculated. Hierarchical clustering [18] was then employed to create a network that visualizes the clustering relationships. The Kaplan-Meier analysis (K-M) displayed the decreased survival rate free from composite endpoints, and comparison of prognosis was performed using log-rank test. The generalized linear mixed-effects models (GLMM) were constructed to analyze the relationship between repeatedly measured urinary albumin/creatinine ratio, serum creatinine and albumin in the two cohorts [19]. The GLMM accounts for random effects (including individual disparities), and comparisons were performed using Analysis of Variance (ANOVA). Line charts were plotted to present adjusted marginal means [20] and standard errors (SEs) of urinary albumin/creatinine ratio, serum creatinine and albumin and illustrate the trend more directly. Multivariate-Cox-proportional-hazards models were employed to determine independent factors associated with the outcomes. The models were constructed from factors with statistical differences in univariate Cox regression analysis, using forward-backward stepwise methods and Akaike information criterion for optimization. All reported p-values were two-tailed, and a significance level of *p* < 0.05 was considered statistically significant. Multiple imputations was used to process the missing value [21]. The data analysis and visualization were performed using R v4.1.3 [22] and R packages (such as *gtools, nlme,* and *surveminer*).

## Results

### Baseline characteristics

**Figure 1.**
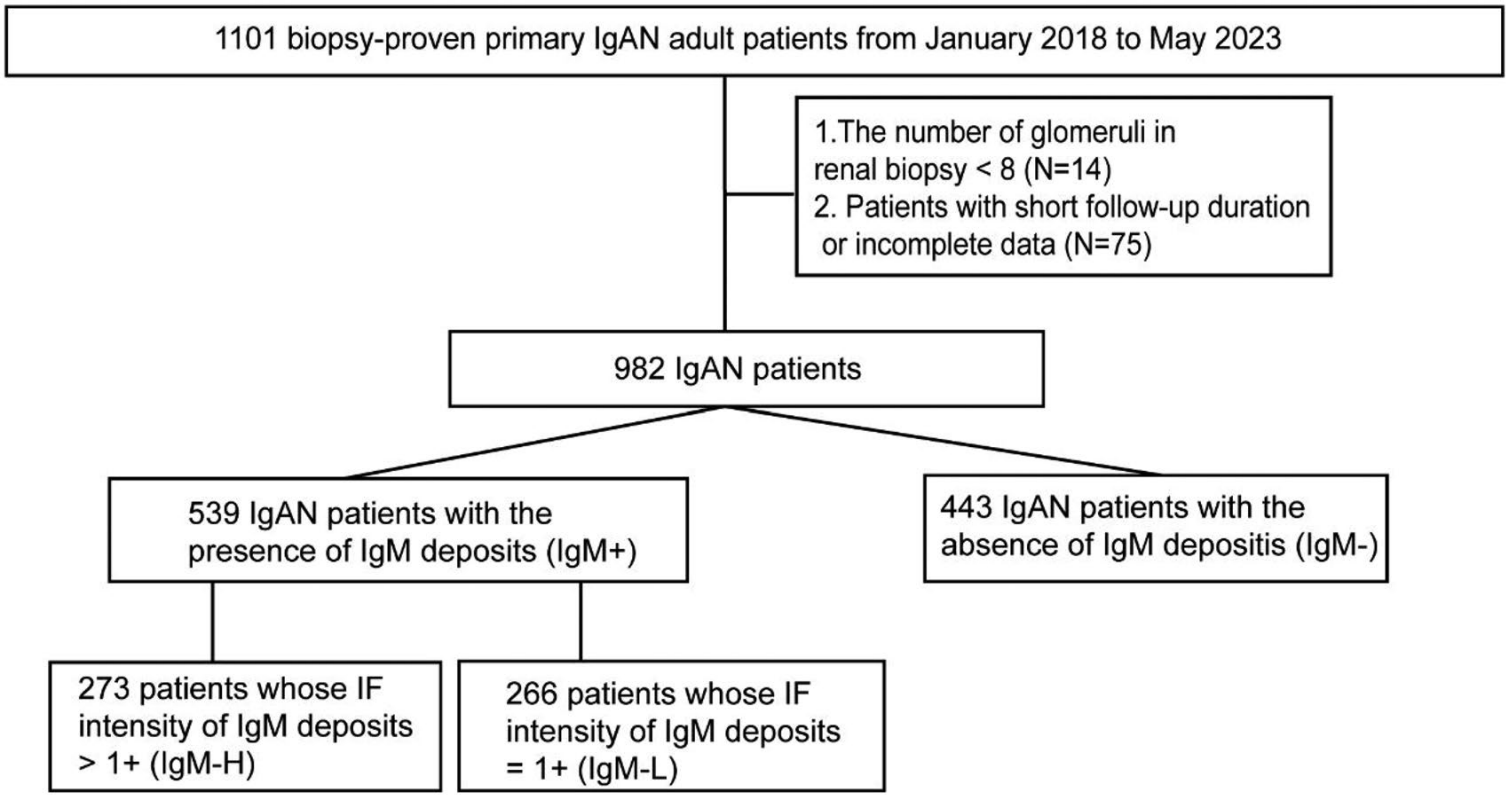
Flowchart of case ascertainment.

From January 2018 and May 2023, there were 1101 biopsy-proven primary IgAN cases at our center (Figure 1). A total of 119 patients were excluded due to short follow-up time, inadequate data or insufficient glomerular count, and the remaining 982 cases were ultimately recruited into this research. Among them, 539 IgAN patients were included in the IgM + cohort due to the positive glomerular IgM deposits in their renal IF. Subsequently, the IgM + cohort was matched with 443 IgAN patients with the absence of glomerular IgM deposits (IgM– cohort), in a 1:1 ratio using PSM. The results of disparities between the two cohort are shown in Table S1. Following the PSM process, which accounted for gender, age, follow-up time and eGFR, no obvious differences were observed in the demographic variables between the IgM+ (left-column) and IgM– (right-column). The IgM + had significantly decreased levels of median albumin (*p* = 0.04) and HDL-cholesterol (*p* = 0.04). The median serum IgG (*p* = 0.04) was level was remarkably higher in IgM+. Additionally, the IF intensities of glomerular IgA (*p* < 0.01), IgG (*p* = 0.02) and C3 (*p* < 0.01) deposits in the IgM + were significantly stronger. These disparities were also observed between the two cohorts before matching (Table S2).

There were 273 cases in the IgM + cohort with the IF intensity of IgM deposits exceeding 1+ (IgM-H), while the remaining cases presented the intensity of 1+ (266 IgM-L). The IgM-H cohort was then matched with the IgM-L cohort in a 1:1 ratio using PSM. The findings presented in Table 1 highlight the differences between the IgM-H (left-column) and IgM-L (right-column) cohort. There were no significant differences in the demographic characteristics between the two cohorts. The IgM-H exhibited remarkably increased levels of median SII (*p* < 0.01), serum IgM (*p* < 0.01), serum IgG (*p* < 0.01), serum C4 (*p* = 0.03) and 24h-UP (*p* = 0.02), UACR (*p* < 0.01) and TA UACR (*p* < 0.01). The median SBP (*p* < 0.01), DBP (*p* < 0.01), Alb (*p* = 0.04), and LDL-cholesterol (*p* < 0.01) were significantly lower in IgM-H. The IgM-H exhibited markedly increased proportion of E1 (*p* = 0.02) and S1 (*p* = 0.02), and decreased proportion of M1 (*p* < 0.01) under the LM. The IF results showed that the intensities of IgA (*p* < 0.01) and C3 (*p* = 0.03) deposits were stronger in the IgM-H. A higher proportion of patients in the IgM-H received Immunosuppressant treatment (*p* = 0.02). Composite endpoints were observed in 30 cases, including 23 IgM-H cases and 7 IgM-L cases, and the incidence was remarkably different between the two cohorts. These differences were also observed between the two cohorts before matching (Table S3).

**Table 1.** baseline characteristics of patients according to the degree of IgM deposits in renal biopsy after matching.

Variables	IgM-H	IgM-L	*p*
*N*	266	266	
Male	96 (36)	107 (40)	0.43
Age, y	41 (29, 49)	39 (32, 49)	0.82
Systolic blood pressure, mmHg	116.49 (108.29, 127.81)	121.33 (112.58, 132.22)	< 0.01
Diastolic blood pressure, mmHg	73.1 (67.17, 77.62)	76 (69.01, 83)	< 0.01
Body mass index	22.68 (20.67, 25.24)	23.46 (20.81, 25.81)	0.31
Hemoglobin, g/L	125 (114, 138)	126 (115.12, 141)	0.33
Serum albumin, g/L	35.7 (33.3, 38.7)	36.7 (33.9, 39.85)	0.04
Serum creatinine, mmol/L	84.5 (66, 110)	81 (64, 109.75)	0.39
eGFR	83.84 (63.83, 106.81)	90.09 (63.43, 111.81)	0.31
Uric acid, mmol/L	360.32 ± 92.26	362.62 ± 93.2	0.77
Total cholesterol, mmol/L	4.92 (4.24, 5.82)	5 (4.33, 5.78)	0.72
Triglyceride, mmol/L	1.63 (1.18, 2.4)	1.62 (1.08, 2.21)	0.21
HDL-cholesterol, mmol/L	1.06 (0.88, 1.3)	1.11 (0.95, 1.32)	0.06
LDL-cholesterol, mmol/L	2.79 (2.2, 3.32)	3 (2.54, 3.6)	< 0.01
SII	6.21 (4.49, 8.05)	5.18 (3.95, 7.76)	<0.01
Serum IgA, g/L	3.12 (2.52, 3.88)	3.2 (2.55, 3.95)	0.81
Serum IgM, g/L	10.8 (9.18, 12.8)	10.1 (7.94, 12.28)	< 0.01
Serum IgG, g/L	1.25 (0.87, 1.57)	1.06 (0.71, 1.47)	< 0.01
Serum C3, g/L	1.07 (0.94, 1.23)	1.06 (0.89, 1.21)	0.08
Serum C4, g/L	0.25 (0.2, 0.3)	0.23 (0.19, 0.28)	0.03
24h urinary protein, g/d	1.11 (0.56, 2.48)	0.95 (0.43, 1.82)	0.02
UACR, mg/g	491.46 (232.27, 1020.77)	324.94 (114.1, 905.83)	<0.01
Treatment			
RAS blocker	225 (85)	229 (86)	0.73
Glucocorticoid	140 (53)	126 (47)	0.29
Immunosuppressant	78 (29)	53 (20)	0.02
Cyclophosphamide	16 (6.0)	17 (6.4)	0.97
Mycophenolate Mofetil	19 (7.1)	17 (6.4)	0.91
Tripterygium Wilfordii	47 (17.7)	26 (9.8)	<0.01
Pathology			
M1	84 (44)	128 (60)	<0.01
E1	98 (51)	79 (37)	<0.01
S1	146 (76)	138 (65)	0.02
T-score			0.07
1	51 (26)	42 (20)	
2	16 (8)	10 (5)	
C1	148 (56)	143 (55)	0.98
IgA			< 0.01
1+	1 (0.4)	1 (0.4)	
2+	11 (4)	24 (9)	
3+	179 (67)	205 (77)	
4+	75 (28)	36 (14)	
IgM			< 0.01
1+	0 (0)	266 (100)	
>1+	266 (100)	0 (0)	
IgG			0.31
1+	11 (4)	16 (6)	
2+	23 (9)	13 (5)	
3+	3 (1)	3 (1)	
C3			0.04
1+	1 (0.4)	1 (0.4)	
2+	11 (5)	24 (9)	
3+	254 (95)	241 (91)	
C4(1+)	5 (2)	0 (0)	0.06
Follow up			
Follow-up duration, m	34.97 (19.1, 48.6)	28.43 (16.03, 48.63)	0.22
TA SII	5.38 (3.98, 7.39)	5.13 (3.85, 6.68)	0.18
TA UACR, mg/g	411.31 (183.24, 916.87)	264.97 (106.07, 717.45)	<0.01
Composite endpoint	23 (9)	7 (3)	<0.01

Numerical variables are reported as mean ± SD or median (IQR), while categorical variables are presented as counts (%).

IgM-H refers to IgAN patients whose IF intensity of IgM deposits exceeded 1+, while IgM-L represents IgAN patients whose IF intensity of IgM deposits was equal to 1+. The composite endpoint was referred to a continuous decline in estimated eGFR from baseline of ≥50% or reaching ESRD.

E; endocapillary hypercellularity; M1; mesangial hypercellularity; T1-2: interstitial fibrosis/tubular atrophy; S: segmental glomerulosclerosis/adhesion; C1: crescent; UACR: urine albumin/creatinine ratio; SII: systemic immune-inflammation index; TA UACR: time averaged urine albumin/creatinine ratio; TA SII: time averaged immune-inflammation index.

*p*-value was adjusted for multiple comparisons.

**Figure 2.**
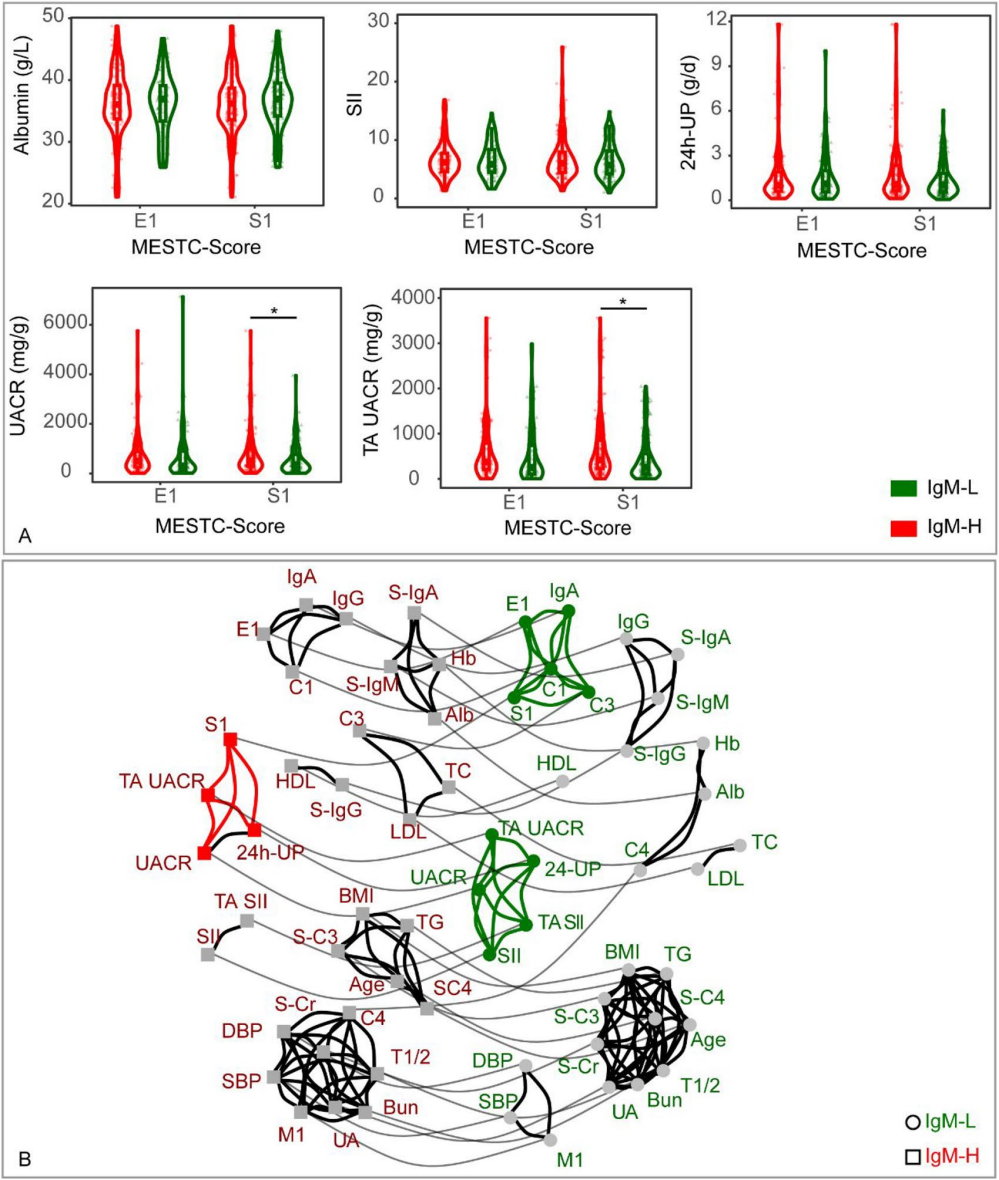
(A) Disparities in clinical data between the IgM-L and IgM-H in different subgroups. The subgroups were constructed according to the pathologic manifestation in the Oxford classification. The medians, interquartile ranges, and distributions of clinical data in each subgroup were calculated to perform the violin, scatter, and box plots. The *p* value <0.05 was marked as ‘*’ and *p* < 0.01 as ‘**’ in the plot. (B) The network diagram illustrates the clustering relationship of clinical factors within IgM-L and IgM-H. All linear data were converted to hierarchical data according to the quantile. Each factor is represented by a node, with circle nodes representing IgM-H and square nodes representing IgM-L. Nodes that are linked by solid black lines indicate a stronger correlation and form a cluster, while nodes that are linked by solid gray lines represent the same factors from various groups (assigned a small weight for the same factors and a larger weight for different factors). IgM-H refers to IgAN patients whose IF intensity of IgM deposits exceeded 1+, while IgM-L represents IgAN patients whose IF intensity of IgM deposits was equal to 1+. Alb: serum albumin; BMI: body mass index; Hb: hemoglobin; HDL: HDL-cholesterol; LDL: LDL-cholesterol; SBP: systolic blood pressure; DBP: diastolic blood pressure; S-Cr: serum creatinine; TC: total cholesterol; TG: triglyceride; UA: serum uric acid; S-IgA: serum IgA; S-IgG: serum IgG; S-IgM: serum IgM; S-C3: serum C3; S-C4: serum C4; UACR: urine albumin/creatinine ratio; TA UACR: time averaged urine albumin/creatinine ratio; SII: systemic immune-inflammation index; TA SII: time averaged systemic immune-inflammation index; 24h-UP: 24-hour urine protein.

The violin plots (Figure 2(A)) revealed that the IgM-H had significantly increased UACR and TA UACR levels in the S1 subgroup (*p* < 0.05) than the IgM-L. Noticeable differences were observed in the cluster network diagram (Figure 2(B)), in which the segmental glomerulosclerosis was correlated with the IgM-H and IgM-L cohorts. Segmental glomerulosclerosis in the IgM-L cohort was correlated with endocapillary hyper cellularity, crescents, IgA and C3 deposits. Nevertheless, in the IgM-H cohort, segmental glomerulosclerosis was correlated with UACR, TA UACR and 24-UP. The Urine protein was correlated with SII in the IgM-L cohort. Conversely, in the IgM-H cohort, the Urine protein was correlated with segmental glomerulosclerosis.

### Prognostic outcomes and risk factors

The median follow-up time of patients selected was 31.9 months, ranging from 6 to 64 months. There were no obvious differences in baseline demographic variables and baseline renal function between the following matching cohorts.

**Figure 3.**
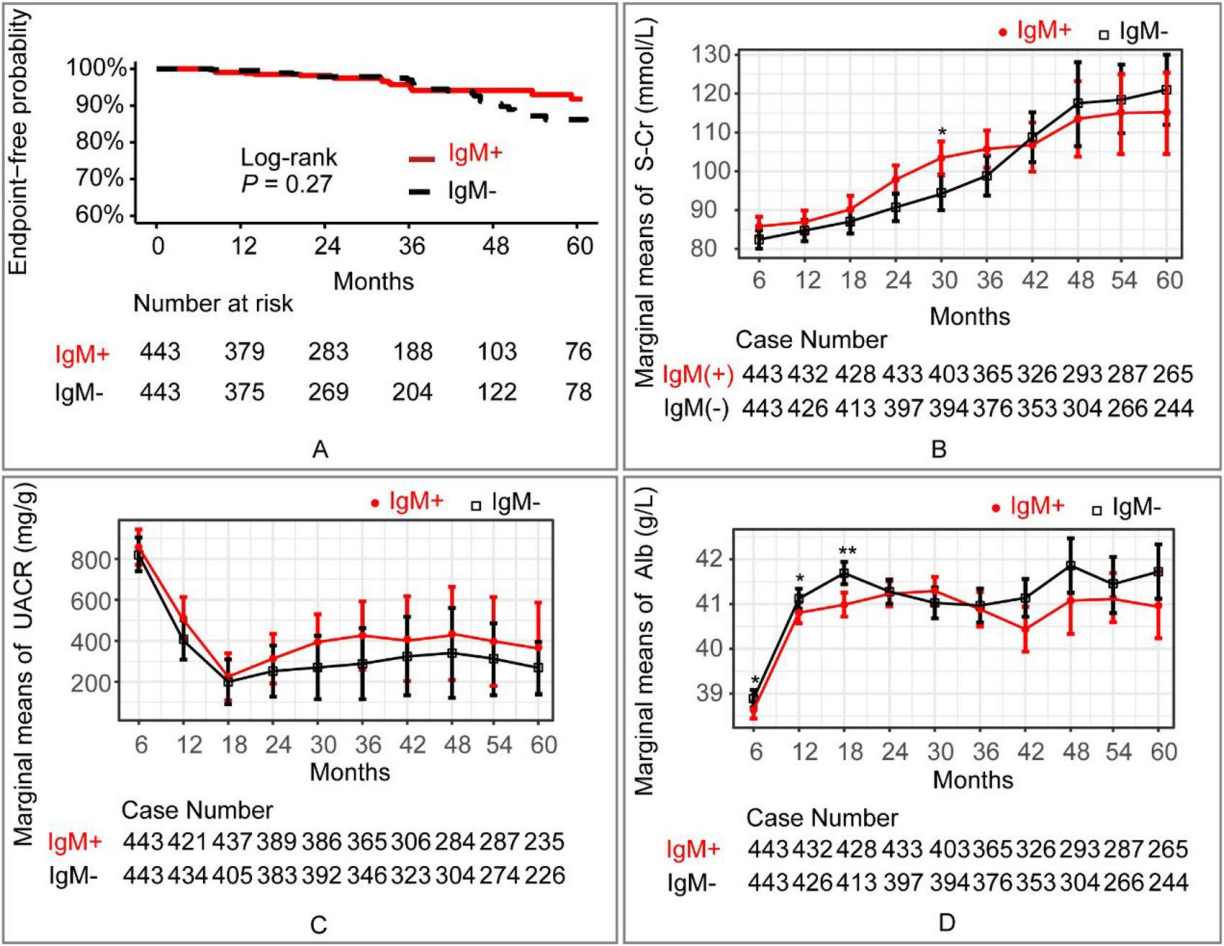
(A) Kaplan-Meier Curve displayed the prognosis of the IgM + and IgM– cohorts (*p* = 0.27 [log-rank test]). The endpoint was defined as either a reduction of ≥50% in baseline eGFR or the development of ESRD. (B) Estimated marginal means and corresponding SEs of serum creatinine levels were estimated and compared between IgM + and IgM– cohorts within the follow-up period using GLMM. (C) Estimated marginal means and corresponding SEs of urine albumin/creatinine ratio levels were estimated and compared between IgM + and IgM– cohorts within the follow-up period using GLMM. (D) Estimated marginal means and corresponding SEs of serum albumin levels were estimated and compared between IgM + and IgM– cohorts within the follow-up period using GLMM. Statistical significance levels are indicated in the plot as ‘*’, which represents *p* < 0.05, and ‘**’, representing *p* < 0.01. The ANOVA results of the GLMM are presented in Table S6. IgM + refers to IgAN patients with the presence of IgM deposits. IgM– refers to IgAN patients with the absence of IgM deposits. Alb: serum albumin; S-Cr: serum creatinine; UACR: urine albumin/creatinine ratio.

The KM curve (Figure 3(A)) displayed no obvious differences in the survival rate without composite endpoints between the IgM + and IgM- cohorts (Log-rank, *p* = 0.27). The line chart of the marginal means (Figure 3(B/C/D)) indicated a clear decrease in UACR and an increase in serum albumin and creatinine in both cohorts during the follow-up period. The GLMM (Table S4) revealed that no significant differences of UACR, serum albumin and creatinine were observed between the two cohorts. The between-group variation interacted with the duration of follow-up, as indicated by the line chart, which showed no significant between-group variation at the 5-year follow-up. In the multivariate Cox regression analysis (Table S5), increased mean arterial pressure (*p* < 0.01), increased uric acid (*p* < 0.01) and presence of T2 (interstitial fibrosis/tubular atrophy > 50%) (*p* < 0.01) were identified as independent risk factors.

**Figure 4.**
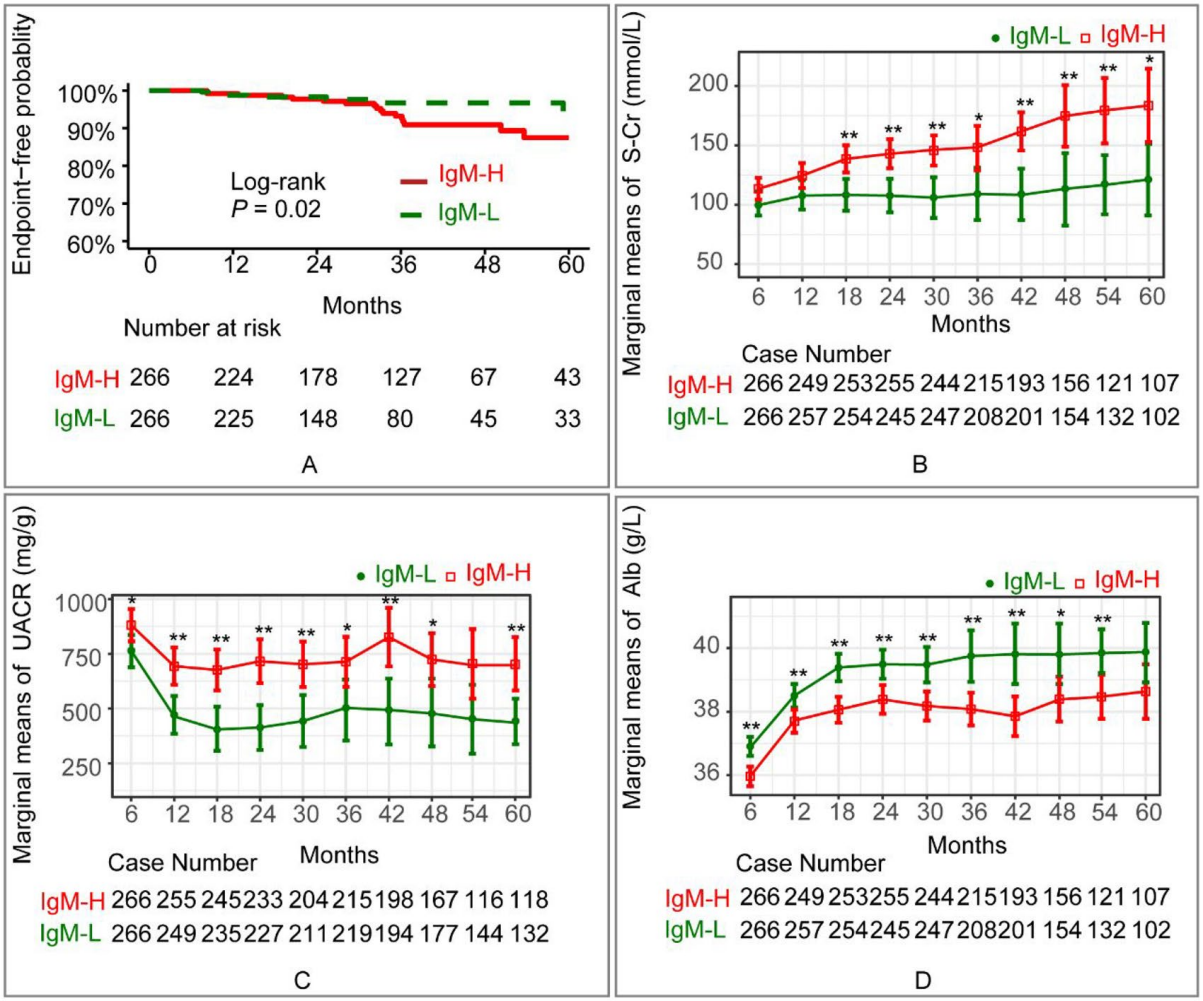
(A) Kaplan-Meier curve displayed the prognosis of the IgM-L and IgM-H cohorts (*p* = 0.02 [log-rank test]). The endpoint was defined as either a reduction of ≥50% in baseline eGFR or the development of ESRD. (B) Estimated marginal means and corresponding SEs of serum creatinine levels were estimated and compared between IgM-L and IgM-H cohorts within the follow-up period using GLMM. (C) Estimated marginal means and corresponding SEs of urine albumin/creatinine ratio levels were estimated and compared between IgM-L and IgM-H cohorts within the follow-up period using GLMM. (D) Estimated marginal means and corresponding SEs of serum albumin levels were estimated and compared between IgM-L and IgM-H cohorts within the follow-up period using GLMM. Statistical significance levels are indicated in the plot as ‘*’, which represents *p* < 0.05, and ‘**’, representing *p* < 0.01. The ANOVA results of the GLMM are presented in Table S7. IgM-H refers to IgAN patients whose IF intensity of IgM deposits exceeded 1+, while IgM-L represents IgAN patients whose IF intensity of IgM deposits was equal to 1+. Alb: serum albumin; S-Cr: serum creatinine; UACR: urine albumin/creatinine ratio.

Notably, the KM (Figure 4(A)) curve demonstrated a remarkably lower survival rate without composite endpoints in the IgM-H than the IgM-L (log-rank, *p* = 0.02). In the multivariate Cox regression analysis (Table 2), the weaker IF intensity of IgM deposit (*p* = 0.03) was found to be an independent protective factor while increased mean arterial pressure (*p* < 0.01), increased serum creatinine (*p* < 0.01) and increased TA UACR (*p* < 0.01) were identified as independent risk factors. In the final model (Table 3-Model4) in which intensity of glomerular IgM deposits was adjusted for Oxford renal pathological classification (MESTC), intensity of glomerular IgA, IgG and C3 deposit, and therapy, increased intensity of glomerular IgM deposits was a significant predictor of poor renal outcome in IgM+. The line chart of the marginal means (Figure 4(B–D)) and the GLMM (Table S6) displayed a significant decrease in UACR and an increase in serum albumin and creatinine in both cohorts during the follow-up period, with significant differences between the two cohorts (UACR: *p* < 0.01; serum creatinine: *p* = 0.04; serum albumin: *p* < 0.01). The between-group variation interacted with the duration of follow-up, as indicated by the line chart, which showed no significant between-group variation at the 5-year follow-up.

**Table 2.** Factors associated with reaching the composite endpoint in matched IgM-L and IgM-H cohort.

Variable	HR	95% CI	*p*	HR	95% CI	*p*
Age	0.97	0.93–1	0.03	0.97	0.94–1.01	0.21
Male	1.78	0.86–3.69	0.1			
BMI	1.00	1–1.01	0.1			
MAP	1.53	1.24–1.88	<0.01	1.54	1.12–2.13	<0.01
Hemoglobin	0.78	0.63–0.95	0.02			
Albumin	0.90	0.85–0.96	<0.01	0.99	0.9–1.08	0.82
Serum creatinine	3.89	2.8–5.43	<0.01	3.05	1.87–4.99	<0.01
Uric acid	1.29	1.03–1.62	0.03			
Total cholesterol	1.21	0.96–1.51	0.1			
Triglyceride	1.18	1–1.38	0.04	0.77	0.54–1.11	0.22
HDL-cholesterol	0.17	0.04–0.8	0.03			
LDL-cholesterol	1.39	1–1.95	0.06			
TA SII	1.03	0.93–1.14	0.5			
TA UACR	1.13	1.1–1.17	<0.01	1.15	1.1–1.2	<0.01
24h-UP	1.39	1.26–1.53	<0.01			
M1	4.61	1.29–16.51	0.02			
E1	1.44	0.52–3.98	0.5			
S1	1.37	0.44–4.27	0.6			
T-score						
T0	Reference	Reference	–			
T1	7.17	1.39–27.08	0.02			
T2	9.19	2.8–32.22	<0.01			
C1	0.84	0.42–1.74	0.6			
IF intensity of IgA	1.47	0.92–4.13	0.9			
IF intensity of IgG	0.64	0.32–2.35	0.8			
IF intensity of C3	1.44	0.43–1.86	0.9			
Serum IgA	0.89	0.62–1.29	0.5			
Serum IgM	0.88	0.78–0.99	0.03			
Serum IgG	1.51	0.86–2.65	0.2			
Serum C3	1.54	0.3–8.02	0.6			
Serum C4	0.82	0.5–1.98	0.1			
Decreased intensity of IgM deposits	0.39	0.17–0.92	0.03	0.38	0.16–0.90	0.03
Glucocorticoid	1.14	0.54–2.39	0.7			
Immunosuppressant	1.18	0.54–2.61	0.7			
RAS blocker	0.67	0.27–1.64	0.4			

IgM-H refers to IgAN patients whose IF intensity of IgM deposits exceeded 1+, while IgM-L represents IgAN patients whose IF intensity of IgM deposits was equal to 1+. Composite endpoint was defined as eGFR decreasing from the baseline ≥50% continuously or reaching ESRD. Factors with statistical differences (*p* < 0.1) in univariate Cox regression were selected to build a multivariate Cox regression model. Then, the AIC using the forward-backward stepwise was performed to select the optimal model with the lowest AIC value. BMI: body mass index; TA UACR: time averaged urine albumin/creatinine ratio; TA SII: time averaged systemic immune-inflammation index; MAP: mean arterial pressure; M1: mesangial hypercellularity; E1: endocapillary hypercellularity; S1: segmental glomerulosclerosis/adhesion; T1-2: the severity of tubular atrophy/interstitial fibrosis; C1: presence of crescent; HR: hazard ratio; CI: confidence interval; AIC: Akaike information criterion; 24h-UP: 24-h urine protein.

**Table 3.** Association between intensity of glomerular IgM deposit and composite endpoint (multivariate Cox regression model).

Variables	Model1		Model2		Model3		Model4	
	HR (95%CI)	*p*	HR (95%CI)	*p*	HR (95%CI)	*p*	HR (95%CI)	*p*
Intensity of IgM deposit								
1+	Ref		Ref		Ref		Ref	
>1+	2.63 (1.11–6.25)	0.03	2.31 (1.04–6.66)	0.04	2.32 (1.04–6.64)	0.04	2.57 (1.07–5.68)	0.02

Composite endpoint was defined as eGFR decreasing from the baseline ≥50% continuously or reaching ESRD. HR: Hazard Ratio; CI: Confidence Interval; M1: mesangial hypercellularity; E1: endocapillary hypercellularity; S1: segmental glomerulosclerosis/adhesion; T1-2: the severity of tubular atrophy/interstitial fibrosis; C1: presence of crescent.

Model1: Adjust for gender, age, follow-up time and eGFR.

Model2: Model1 + Adjust for M, E, S, T, C.

Model3: Model2 + Adjust for glomerular IgA, IgG, C3 deposits.

Model4: Model3 + Adjust for use of glucocorticoid, immunosuppressant and RAS blocker.

The prognostic outcomes of the IgM– were also compared with those of the IgM-L. The KM curve (Figure S2(A)) displayed no obvious differences in the survival rate without composite endpoints between the two cohorts (Log-rank, *p* = 0.1). The line chart of the marginal means (Figure S2(B–D)) indicated a clear decrease in UACR and an increase in serum albumin and creatinine in both cohorts during the follow-up period. The GLMM (Table S7) revealed that no significant differences of UACR, serum albumin and creatinine were observed between the two cohorts. The between-group variation interacted with the duration of follow-up, as indicated by the line chart, which showed no significant between-group variation at the 5-year follow-up.

## Discussion

Previously, it was reported that approximately 25–80% of IgAN patients presented with positive glomerular IgM deposits (referred to as IgM+) [4,6,9]. In our retrospective analysis of 982 IgAN patients, we identified 539 patients with IgM+ (54.9%), which was consistent with previous research results. IgM + patients exhibited lower serum albumin levels as well as stronger intensity of IgA and C3 at biopsy. These findings suggest a more severe disease activity and worse prognosis, which was found in previous studies [23–25]. Surprisingly, when utilizing a composite endpoint of eGFR decline of ≥50% from baseline or reaching ESRD, our KM analysis revealed no obvious difference in prognosis between the IgM + and IgM- cohorts after matching gender, age, follow-up time, and eGFR at baseline. Additionally, multivariate Cox regression analysis showed that glomerular IgM deposit was not an independent factor associated with the endpoint. These findings are in disagreement with some prior studies [6,7], which demonstrated that glomerular IgM deposit contributed adversely to the outcome. Such inconsistent results may be ascribed to the different definitions of the outcome. Thus, we speculate that the changes in urine protein, serum albumin and creatinine during the follow-up may hold great significance for understanding the prognostic features. However, the GLMM and line charts demonstrated an insignificant between-group variation of urine albumin/creatinine ratio, serum albumin and creatinine during the follow-up, suggesting that the outcomes did not differ significantly between the two cohorts. The larger sample size and long follow-up time also further improve the accuracy of the results.

Tan et al. [6] proposed in their study that the different degrees of mesangial IgM deposition have a negative correlation with histological activity, clinical severity and renal outcome. Thus, we subsequently categorized IgM + patients into two cohorts according to the intensity of IgM deposits (referred to as IgM-H and IgM-L), and analyzed their disparities. Although there were no obvious disparities in age, gender, follow-up time, and eGFR between the two groups, we still perform the PSM to further narrow the differences. Notably, KM analysis revealed that the prognostic outcomes of IgM-H were obviously worse than those of IgM-L. The GLMM and line chart demonstrated a remarkable higher urine albumin/creatinine ratio and serum creatinine levels as well as lower serum albumin level in IgM-H persistently, suggesting the worse outcome of IgM-H. IgM-H patients presented with higher urine protein, SII, serum IgM, IgG, C4 level as well as lower serum albumin level. In addition, more IgM-H patients received immunosuppressant therapy. These findings suggest a more aggressive inflammatory response and potentially stronger immune-inflammatory reactions in IgM-H patients, which was proposed in prior studies [10,14,26]. Previous animal experiment showed that IgM antibodies bind to glomerular epitopes and that IgM contributes to the progression of glomerular damage [27]. Stefan et al. [7] did not observe a relationship between the presence or intensity of mesangial IgM deposits and the Oxford MEST-C classes. Our study found that except for stronger intensity of IgA and C3 deposits, IgM-H exhibited more pronounced glomerular endocapillary hypercellularity and segmental glomerulosclerosis/adhesion, which were considered as independent risk factors of IgAN in previous study [1]. To explore association between the clinical indicators and pathologic manifestation, we performed the subgroup analysis by Oxford MEST-C classification for the indicators with between-group variations. Interestingly, IgM-H presented with significantly higher urine protein level while accompanied by segmental glomerulosclerosis/adhesion. Previous studies indicated the usual association of IgM deposition in segmental sclerosis with a higher grade of chronicity in the kidney [4,9]. Therefore, it was necessary to explore the significance of segmental glomerulosclerosis/adhesion and urine protein in IgAN with different IgM deposits intensities. Our cluster analysis revealed that the correlation patterns of S1 differed between the IgM-H and IgM-L, suggesting that segmental glomerulosclerosis/adhesion may have distinct clinical significance in each cohort. In IgM-H, the segmental glomerulosclerosis/adhesion may correlate with urine protein, which was a well-known risk factor associated with poorer outcome [28]. In contrast, in IgM-L, the segmental glomerulosclerosis/adhesion may correlate with some pathological manifestations while urine protein may correlate with inflammatory indicators. These findings suggest that the segmental glomerulosclerosis/adhesion in IgM-H may associated with the severe clinical manifestations more directly. Thus, we speculated that the IgM-H and IgM-L may represent two different phases of the disease. Moriyama et al. [9] concluded that although glomerular IgM deposits was associated with certain chronic glomerular lesions. Liu et al. [26] revealed that frequency of IgM deposit in renal glomeruli increased remarkably at the repeated renal biopsies during the course of disease. Our findings could complement these results. Of note, renal survival negatively correlates with higher degree of glomerular IgM deposits independent of Oxford renal pathological classification (MESTC), intensity of glomerular IgA, IgG and C3 deposit, and therapy.

It was interesting that the prognostic difference was observed between the IgM-H and IgM-L, but not between the IgM + and IgM–. Therefore, we attempted to compare the disparities between IgM– and IgM-L. The results displayed that the prognosis of IgM-L was not worse than that of IgM–, suggesting weak intensity of glomerular IgM deposit may not contribute adversely to the outcome. The prognostic difference between IgM– and IgM-L was not well illustrated in previous study [4,6,7]. It was reported previously that IgM can activate the lectin pathway of the complement system and cause inflammation in patients with IgAN [6]. According to the baseline characteristics of patients in IgM– and IgM-L cohort after matching (Table S8), we speculate that weak intensity of IgM deposits may not induce severe inflammation enough to cause renal damage. On the other hand, nonspecific glomerular IgM deposits may be included in the IgM-L. Further studies were required. The proportions of IgM-L and IgM-H in IgM + in previous research were also different from ours or unclear, which may account for the conflicting conclusion. Moreover, racial differences and potential nonspecific glomerular IgM deposits may also influence the results, requiring more multi-center studies.

## Conclusion

To summarize, our research offered further findings on the clinical significance of glomerular IgM deposits in patients with IgAN. Notably, the prognosis of IgM + was not markedly worse compared to IgM–. In IgAN patients with positive IgM deposits, however, increased intensity of glomerular IgM deposits may contribute adversely to clinicopathologic presentation and the outcome, with persistently lower serum albumin, higher urine albumin/creatinine ratio and serum creatinine levels during the follow-up. Decreased intensity of IgM deposits was determined as an independent protective factor in those patients. Nevertheless, it is a retrospective single-center study. Moreover, it was challenging to obtain detailed therapy regimens and long-term follow-up data. The GLMMs were constructed to eliminate the impact of confounding factors such as long-term follow-up and individual disparities yet it was hard to reduce the all such effects. Consequently, the conclusions drawn here necessitate further confirmation through studies conducted across multiple centers.

## Supplementary Material

Supplemental materials.pdf

## Data Availability

Data is not publicly available due to ethical reasons. Further enquiries can be directed to the corresponding author.
