# Supplementary material for: Clinical significance of glomerular IgM deposit in IgA nephropathy: a 5-year follow-up study
Source: Ren Fail. 2024 Aug 1;46(2):2386146. doi: 10.1080/0886022X.2024.2386146 (PMC11299447; doi:10.1080/0886022X.2024.2386146)
Supplement: Supplemental materials.pdf [file IRNF_A_2386146_SM6299.pdf]

TableS1 Baseline characteristics of patients according to the presence or absence of IgM deposition in renal biopsy after matching

Variables	IgM+	IgM-	*P*
N	443	443	
Male	190 (43)	198 (45)	0.61
Age, y	40 (31, 48.5)	39 (30, 48.5)	0.54
Systolic blood pressure, mmHg	118.25 (110.31, 130)	119.5 (110.77, 131.09)	0.29
Diastolic blood pressure, mmHg	74 (67.78, 79.61)	74.33 (68.78, 80.11)	0.18
Body mass index	23.12 (20.81, 25.42)	22.86 (20.9, 24.99)	0.73
Hemoglobin, g/L	127.5 ± 19.53	128.73 ± 19.94	0.42
Serum albumin, g/L	36.5 (33.45, 39.15)	37.1 (33.7, 39.9)	0.04
Serum creatinine, mmol/L	87 (67, 112.25)	88 (69, 111)	0.62
eGFR	86.21 (62.97, 108.9)	86.86 (62.67, 108.29)	0.76
Uric acid, mmol/L	354 (302, 429)	356 (288.5, 424.5)	0.64
Total cholesterol, mmol/L	4.99 (4.3, 5.79)	4.91 (4.22, 5.66)	0.23
Triglyceride, mmol/L	1.64 (1.16, 2.33)	1.64 (1.16, 2.37)	0.71
HDL-cholesterol, mmol/L	1.05 (0.91, 1.26)	1.08 (0.93, 1.31)	0.04
LDL-cholesterol, mmol/L	2.89 (2.33, 3.45)	2.77 (2.35, 3.38)	0.22
SII	5.75 (4.31, 7.93)	5.82 (4.27, 8.03)	0.58
Serum IgA, g/L	3.13 (2.53, 3.92)	3.26 (2.52, 4.17)	0.21
Serum IgM, g/L	10.5 (8.54, 12.6)	10.6 (8.59, 12.7)	0.62
Serum IgG, g/L	1.15 (0.79, 1.5)	1.07 (0.71, 1.41)	0.04
Serum C3, g/L	1.07 (0.91, 1.21)	1.04 (0.92, 1.19)	0.12
Serum C4, g/L	0.24 (0.19, 0.3)	0.24 (0.19, 0.29)	0.39
24h urinary protein, g/d	1.14 (0.54, 2.46)	1.21 (0.62, 2.35)	0.62
UACR, mg/g	376.05 (151.2, 916.45)	361.08 (173.99, 891.8)	0.64
**Treatment**			
RAS blocker	228 (51)	238 (54)	0.49
Glucocorticoid	111 (25)	134 (30)	0.12
Immunosuppressant	115 (26)	134 (30)	0.22
Cyclophosphamide	30 (6.7)	37 (8.4)	0.41
Mycophenolate Mofetil	35 (7.9)	47 (10.6)	0.21
Tripterygium Wilfordii	65 (14.7)	76 (17.2)	0.41
**Pathology**			
M1	179 (53)	155 (50)	0.52
E1	145 (43)	143 (46)	0.53
S1	238 (70)	209 (67)	0.49
T-score			0.62
1	82 (24)	86 (28)	
2	22 (7)	18 (6)	
C1	251 (57)	266 (61)	0.32
IgA			< 0.01
1+	2 (0.5)	55 (12)	
2+	28 (6)	50 (11)	

3+	320 (72)	261 (59)	
4+	93 (21)	77 (17)	
IgM			< 0.01
1+	223 (50)	0 (0)	
>1+	220 (50)	0 (0)	
IgG			0.02
1+	20 (5)	11 (2)	
2+	32 (7)	15 (3)	
3+	6 (1)	7 (2)	
C3			< 0.01
1+	2 (0.5)	55 (12)	
2+	28 (6)	50 (11)	
3+	413 (93)	338 (76)	
C4(1+)	4 (1)	1 (0.2)	0.41
**Follow up**			
Follow-up duration, m	32.47 (17.55, 46.47)	32.43 (16.32, 50.02)	0.83
TA SII	5.33 (3.9, 6.98)	5.34 (3.98, 7.19)	0.42
TA UACR, mg/g	333.91 (124.9, 805.07)	302.27 (146.1, 725.23)	0.71
Composite endpoint	24 (5)	31 (7)	0.44

Numerical variables are reported as mean ± SD or median (IQR), while categorical variables are presented as counts (%).

IgM+ refers to IgAN patients with the presence of IgM deposits. IgM- refers to IgAN patients with the absence of IgM deposits. The composite endpoint was referred to a continuous decline in estimated eGFR from baseline of ≥50% or reaching ESRD.

E, endocapillary hypercellularity; M1, mesangial hypercellularity; T1-2; interstitial fibrosis/tubular atrophy; S, segmental glomerulosclerosis/adhesion; C1, crescent; UACR, urine albumin/creatinine ratio; SII, systemic immune-inflammation index; TA UACR, time averaged urine albumin/creatinine ratio; TA SII, time averaged immune-inflammation index.

P-value was adjusted for multiple comparisons.

TableS2 Baseline characteristics of patients according to the presence or absence of IgM deposition in renal biopsy

Variables	IgM +	IgM-	*P*
N	539	443	
Male	204 (38)	198 (45)	0.04
Age, y	40 (31, 49)	39 (30, 48.5)	0.41
Systolic blood pressure, mmHg	118.72 (110.69, 130)	119.5 (110.77, 131.09)	0.61
Diastolic blood pressure, mmHg	74.67 (67.88, 80)	74.33 (68.78, 80.11)	0.32
Body mass index	23.11 (20.76, 25.45)	22.86 (20.9, 24.99)	0.74
Hemoglobin, g/L	125 (115, 139)	128 (116, 143)	0.08
Serum albumin, g/L	36.4 (33.5, 39.1)	37.1 (33.7, 39.9)	0.03
Serum creatinine, mmol/L	83 (65, 110.5)	88 (69, 111)	0.12
eGFR	86.89 (63.44, 109.48)	86.86 (62.67, 108.29)	0.52
Uric acid, mmol/L	354 (297.75, 428.5)	356 (288.5, 424.5)	0.83
Total cholesterol, mmol/L	4.98 (4.3, 5.8)	4.91 (4.22, 5.66)	0.24
Triglyceride, mmol/L	1.63 (1.15, 2.3)	1.64 (1.16, 2.37)	0.53
HDL-cholesterol, mmol/L	1.04 (0.89, 1.23)	1.08 (0.93, 1.31)	0.02
LDL-cholesterol, mmol/L	2.88 (2.32, 3.45)	2.77 (2.35, 3.38)	0.33
SII	5.8 (4.3, 8.01)	5.82 (4.27, 8.03)	0.62
Serum IgA, g/L	3.14 (2.53, 3.91)	3.26 (2.52, 4.17)	0.21
Serum IgM, g/L	10.6 (8.65, 12.6)	10.6 (8.59, 12.7)	0.68
Serum IgG, g/L	1.16 (0.8, 1.54)	1.07 (0.71, 1.41)	0.02
Serum C3, g/L	1.07 (0.91, 1.23)	1.04 (0.92, 1.19)	0.12
Serum C4, g/L	0.24 (0.19, 0.3)	0.24 (0.19, 0.29)	0.42
24h urinary protein, g/d	1.13 (0.52, 2.38)	1.21 (0.62, 2.35)	0.43
UACR, mg/g	396.56 (160.27, 955.39)	361.08 (173.99, 891.8)	1
**Treatment**			
RAS blocker	460 (85)	371 (84)	0.52
Glucocorticoid	270 (50)	238 (54)	0.32
Immunosuppressant	133 (25)	134 (30)	0.13
Cyclophosphamide	34 (6.3)	37 (8.4)	0.32
Mycophenolate Mofetil	41 (7.6)	47 (10.6)	0.14
Tripterygium Wilfordii	73 (13.5)	76 (17.2)	0.23
**Pathology**			
M1	213 (52)	155 (50)	0.61
E1	178 (44)	143 (46)	0.52
S1	287 (70)	209 (67)	0.54
T-score			0.38
1	95 (23)	86 (28)	
2	26 (6)	18 (6)	
C1	295 (55)	266 (61)	0.07
IgA			< 0.01
1+	2 (0.3)	55 (12)	
2+	35 (6)	50 (11)	

3+	391 (73)	261 (59)	
4+	111 (21)	77 (17)	
IgM			< 0.01
1+	266 (49)	0 (0)	
>1+	273 (51)	0 (0)	
IgG			0.02
1+	27 (5)	11 (2)	
2+	36 (7)	15 (3)	
3+	6 (1)	7 (2)	
C3			< 0.01
1+	2 (0.3)	55 (12)	
2+	35 (6)	50 (11)	
3+	502 (93)	338 (76)	
C4(1+)	5 (1)	1 (0.2)	0.22
**Follow up**			
Follow-up duration, m	30.87 (16.55, 45.55)	32.43 (16.32, 50.02)	0.32
TA SII	5.33 (3.9, 6.98)	5.34 (3.98, 7.19)	0.43
TA UACR, mg/g	333.91 (124.9, 805.07)	302.27 (146.1, 725.23)	0.66
Composite endpoint	31 (6)	31 (7)	0.48

Numerical variables are reported as mean ± SD or median (IQR), while categorical variables are presented as counts (%).

IgM+ refers to IgAN patients with the presence of IgM deposits. IgM- refers to IgAN patients with the absence of IgM deposits. The composite endpoint was referred to a continuous decline in estimated eGFR from baseline of ≥50% or reaching ESRD.

E, endocapillary hypercellularity; M1, mesangial hypercellularity; T1-2; interstitial fibrosis/tubular atrophy; S, segmental glomerulosclerosis/adhesion; C1, crescent; UACR, urine albumin/creatinine ratio; SII, systemic immune-inflammation index; TA UACR, time averaged urine albumin/creatinine ratio; TA SII, time averaged immune-inflammation index.

P-value was adjusted for multiple comparisons.

TableS3 Baseline characteristics of patients according to the degree of IgM deposits in renal biopsy

Variables	IgM-H	IgM-L	*P*
N	273	266	
Male	97 (36)	107 (40)	0.32
Age, y	41 (30, 49)	39 (32, 49)	0.91
Systolic blood pressure, mmHg	116.64 (108.29, 128)	121.33 (112.58, 132.22)	< 0.01
Diastolic blood pressure, mmHg	73.13 (67.33, 77.53)	76 (69.01, 83)	< 0.01
Body mass index	22.6 (20.62, 25.22)	23.46 (20.81, 25.81)	0.21
Hemoglobin, g/L	125 (114, 138)	126 (115.12, 141)	0.23
Serum albumin, g/L	35.8 (33.3, 38.7)	36.7 (33.9, 39.85)	0.04
Serum creatinine, mmol/L	85 (66, 111)	81 (64, 109.75)	0.34
eGFR	83.68 (63.5, 106.32)	90.09 (63.43, 111.81)	0.17
Uric acid, mmol/L	359.43 ± 93.62	362.62 ± 93.2	0.73
Total cholesterol, mmol/L	4.96 (4.25, 5.84)	5 (4.33, 5.78)	0.83
Triglyceride, mmol/L	1.64 (1.19, 2.41)	1.62 (1.08, 2.21)	0.12
HDL-cholesterol, mmol/L	1.05 (0.89, 1.3)	1.11 (0.95, 1.32)	0.06
LDL-cholesterol, mmol/L	2.8 (2.22, 3.35)	3 (2.54, 3.6)	< 0.01
SII	6.2 (4.51, 8.07)	5.18 (3.95, 7.76)	<0.01
Serum IgA, g/L	3.12 (2.52, 3.89)	3.2 (2.55, 3.95)	0.90
Serum IgM, g/L	10.8 (9.19, 12.9)	10.1 (7.94, 12.28)	< 0.01
Serum IgG, g/L	1.25 (0.87, 1.57)	1.06 (0.71, 1.47)	< 0.01
Serum C3, g/L	1.08 (0.94, 1.24)	1.06 (0.89, 1.21)	0.07
Serum C4, g/L	0.25 (0.2, 0.3)	0.23 (0.19, 0.28)	0.03
24h urinary protein, g/d	1.09 (0.55, 2.48)	0.95 (0.43, 1.82)	0.02
UACR, mg/g	487.95 (228.82, 1018.15)	324.94 (114.1, 905.83)	<0.01
**Treatment**			
RAS blocker	231 (85)	229 (86)	0.71
Glucocorticoid	144 (53)	126 (47)	0.22
Immunosuppressant	80 (29)	53 (20)	0.02
Cyclophosphamide	17 (6.2)	17 (6.4)	0.99
Mycophenolate Mofetil	24 (8.8)	17 (6.4)	0.41
Tripterygium Wilfordii	47 (17.2)	26 (9.8)	0.02
**Pathology**			
M1	85 (43)	128 (60)	<0.01
E1	99 (50)	79 (37)	0.02
S1	149 (76)	138 (65)	0.02
T-score			0.06
1	53 (27)	42 (20)	
2	16 (8)	10 (5)	
C1	152 (56)	143 (55)	0.92
IgA			< 0.01
1+	1 (0.4)	1 (0.4)	
2+	11 (4)	24 (9)	

3+	186 (68)	205 (77)	
4+	75 (27)	36 (14)	
IgM			< 0.01
1+	0 (0)	266 (100)	
>1+	273 (100)	0 (0)	
IgG			0.31
1+	11 (4)	16 (6)	
2+	23 (8)	13 (5)	
3+	3 (1)	3 (1)	
C3			0.03
1+	1 (0.4)	1 (0.4)	
2+	11 (4)	24 (9)	
3+	261 (96)	241 (91)	
C4(1+)	5 (2)	0 (0)	0.06
**Follow up**			
Follow-up duration, m	35.23 (18.47, 49.47)	28.43 (16.03, 48.63)	0.21
TA SII	5.48 (3.98, 7.49)	5.13 (3.85, 6.68)	0.12
TA UACR, mg/g	410.34 (174.46, 886.36)	264.97 (106.07, 717.45)	<0.01
Composite endpoint	24 (9)	7 (3)	<0.01

Numerical variables are reported as mean ± SD or median (IQR), while categorical variables are presented as counts (%).

IgM-H refers to IgAN patients whose IF intensity of IgM deposits exceeded 1+, while IgM-L represents IgAN patients whose IF intensity of IgM deposits was equal to 1+. The composite endpoint was referred to a continuous decline in estimated eGFR from baseline of ≥50% or reaching ESRD.

E, endocapillary hypercellularity; M1, mesangial hypercellularity; T1-2; interstitial fibrosis/tubular atrophy; S, segmental glomerulosclerosis/adhesion; C1, crescent; UACR, urine albumin/creatinine ratio; SII, systemic immune-inflammation index; TA UACR, time averaged urine albumin/creatinine ratio; TA SII, time averaged immune-inflammation index.

P-value was adjusted for multiple comparisons.

Table S4-1 GLMM analysis for repeated measures of serum creatinine between matched IgM+ and IgM- cohorts.

	F-value	*P*
(Intercept)	1035	<0.01
Group	2.1	0.12
Follow-up month	57.5	<0.01
Group*Follow-up month	0.8	0.63

The GLMM was constructed to evaluate the association of repeated measures of serum creatinine between the 2 cohorts and among different periods of follow-up. The significance test was reported using the ANOVA to reveal the intergroup and intragroup differences. Cohorts, the IgM+ and IgM-; cohorts*follow-up duration, the interaction of cohorts and follow-up duration.

Table S4-2 GLMM analysis for repeated measures of urine albumin/creatinine ratio between matched IgM+ and IgM- cohorts.

	F-value	*P*
(Intercept)	1029.2	<0.01
Group	0.9	0.32
Follow-up month	9.1	<0.01
Group*Follow-up month	1.1	0.42

The GLMM was constructed to evaluate the association of repeated measures of albumin/creatinine ratio between the 2 cohorts and among different periods of follow-up. The significance test was reported using the ANOVA to reveal the intergroup and intragroup differences. Cohorts, the IgM+ and IgM-; cohorts*follow-up duration, the interaction of cohorts and follow-up duration.

Table S4-3 GLMM analysis for repeated measures of serum albumin between matched IgM+ and IgM- cohorts.

	F-value	*P*
(Intercept)	601.8	<0.01
Group	0.4	0.51
Follow-up month	19.5	<0.01
Group*Follow-up month	0.5	0.79

The GLMM was constructed to evaluate the association of repeated measures of serum albumin between the 2 cohorts and among different periods of follow-up. The significance test was reported using the ANOVA to reveal the intergroup and intragroup differences. Cohorts, the IgM+ and IgM-; cohorts*follow-up duration, the interaction of cohorts and follow-up duration.

TableS5 Factors associated with reaching the composite endpoint in matched IgM+ and IgM- cohort

Variable	HR	95% CI	*P*	HR	95% CI	*P*
Age	0.99	0.97-1.01	0.3			
Male	1.80	1.06-3.07	0.03			
BMI	1.00	0.98-1.01	0.7			
MAP	1.52	1.3-1.79	<0.01	1.64	1.24-2.19	<0.01
Hemoglobin	0.84	0.74-0.96	0.02			
Albumin	0.90	0.86-0.94	<0.01			
Serum creatinine	3.54	2.7-4.66	<0.01			
Uric acid	1.37	1.16-1.6	<0.01	1.69	1.22-2.33	<0.01
Total cholesterol	1.20	1.01-1.42	0.04			
Triglyceride	1.20	1.06-1.36	<0.01			
HDL-cholesterol	0.19	0.06-0.59	<0.01			
LDL-cholesterol	1.28	0.99-1.65	0.06			
TA SII	1.07	1.01-1.14	0.02	1.08	0.98-1.2	0.13
TA UACR	1.08	1.06-1.1	<0.01			
24h-UP	1.21	1.14-1.29	<0.01			
M1	3.77	1.5-9.49	<0.01			
E1	0.71	0.3-1.64	0.4			
S1	3.52	1.05-11.84	0.04			
T-score						
T0	Reference	Reference	-	Reference	Reference	-
T1	4.68	1.44-10.93	<0.01	3.40	0.88-13.15	0.07
T2	9.02	3.21-18.9	<0.01	7.26	1.01-16.92	<0.01
C1	0.74	0.43-1.28	0.3			
IF intensity of IgA	1.09	0.38-3.08	0.9			
IF intensity of IgG	0.58	0.08-4.27	0.6			
IF intensity of C3	1.09	0.32-3.14	0.8			
Serum IgA	1.00	0.78-1.28	1			
Serum IgM	0.89	0.82-0.97	<0.01	0.90	0.78-1.03	0.12
Serum IgG	0.73	0.43-1.25	0.3			
Serum C3	0.72	0.23-2.23	0.6			
Serum C4	0.83	0.51-1.37	0.6			
Presence of IgM	1.35	0.79-2.31	0.3			
Glucocorticoid	1.15	0.67-1.97	0.6			
Immunosuppressant	1.17	0.67-2.05	0.6			
RAS blocker	0.72	0.36-1.43	0.3			

IgM+ refers to IgAN patients with the presence of IgM deposits. IgM- refers to IgAN patients with the absence of IgM deposits. Composite endpoint was defined as eGFR decreasing from the baseline ≥50% continuously or reaching ESRD. Factors with statistical differences ($p<0.1$) in univariate Cox

regression were selected to build a multivariate Cox regression model. Then, the AIC using the forward-backward stepwise was performed to select the optimal model with the lowest AIC value. BMI, body mass index; TA UACR, time averaged urine albumin/creatinine ratio; TA SII, time averaged systemic immune-inflammation index; MAP, mean arterial pressure; M1, mesangial hypercellularity; E1, endocapillary hypercellularity; S1, segmental glomerulosclerosis/adhesion; T1-2, the severity of tubular atrophy/interstitial fibrosis; C1, presence of crescent; HR, hazard ratio; CI, confidence interval; AIC, Akaike information criterion; 24h-UP, 24-hour urine protein.

Table S6-1 GLMM analysis for repeated measures of serum creatinine between matched IgM-L and IgM-H cohorts.

	F-value	*P*
(Intercept)	379.06	<0.01
Cohorts	1.92	0.04
Follow-up month	2.94	<0.01
Cohorts*Follow-up month	0.85	0.43

The GLMM was constructed to evaluate the association of repeated measures of serum creatinine between the 2 cohorts and among different periods of follow-up. The significance test was reported using the ANOVA to reveal the intergroup and intragroup differences. Cohorts, the IgM-L and IgM-H; cohorts*follow-up duration, the interaction of cohorts and follow-up duration.

Table S6-2 GLMM analysis for repeated measures of urine albumin/creatinine ratio between matched IgM-L and IgM-H cohorts.

	F-value	*P*
(Intercept)	228.6	<0.01
Group	4.7	<0.01
Follow-up month	5.9	<0.01
Group*Follow-up month	0.5	0.82

The GLMM was constructed to evaluate the association of repeated measures of albumin/creatinine ratio between the 2 cohorts and among different periods of follow-up. The significance test was reported using the ANOVA to reveal the intergroup and intragroup differences. Cohorts, the IgM-L and IgM-H; cohorts*follow-up duration, the interaction of cohorts and follow-up duration.

Table S6-3 GLMM analysis for repeated measures of serum albumin between matched IgM-L and IgM-H cohorts.

	F-value	*P*
(Intercept)	428.6	<0.01
Group	3.3	<0.01
Follow-up month	21.8	<0.01
Group*Follow-up month	0.9	0.48

The GLMM was constructed to evaluate the association of repeated measures of serum albumin between the 2 cohorts and among different periods of follow-up. The significance test was reported using the ANOVA to reveal the intergroup and intragroup differences. Cohorts, the IgM-L and IgM-H; cohorts*follow-up duration, the interaction of cohorts and follow-up duration.

Table S7-1 GLMM analysis for repeated measures of serum creatinine between matched IgM-L and IgM- cohorts.

	F-value	*P*
(Intercept)	707.11	<0.01
Cohorts	0.57	0.51
Follow-up month	4.81	<0.01
Cohorts*Follow-up month	0.86	0.22

The GLMM was constructed to evaluate the association of repeated measures of serum creatinine between the 2 cohorts and among different periods of follow-up. The significance test was reported using the ANOVA to reveal the intergroup and intragroup differences. Cohorts, the IgM-L and IgM-; cohorts*follow-up duration, the interaction of cohorts and follow-up duration.

Table S7-2 GLMM analysis for repeated measures of urine albumin/creatinine ratio between matched IgM-L and IgM- cohorts.

	F-value	*P*
(Intercept)	388.36	<0.01
Group	2.47	0.13
Follow-up month	11.87	<0.01
Group*Follow-up month	0.77	0.57

The GLMM was constructed to evaluate the association of repeated measures of albumin/creatinine ratio between the 2 cohorts and among different periods of follow-up. The significance test was reported using the ANOVA to reveal the intergroup and intragroup differences. Cohorts, the IgM-L and IgM-; cohorts*follow-up duration, the interaction of cohorts and follow-up duration.

Table S7-3 GLMM analysis for repeated measures of serum albumin between matched IgM-L and IgM- cohorts.

	F-value	*P*
(Intercept)	640.17	<0.01
Group	1.19	0.32
Follow-up month	34.83	<0.01
Group*Follow-up month	1.14	0.31

The GLMM was constructed to evaluate the association of repeated measures of serum albumin between the 2 cohorts and among different periods of follow-up. The significance test was reported using the ANOVA to reveal the intergroup and intragroup differences. Cohorts, the IgM-L and IgM-; cohorts*follow-up duration, the interaction of cohorts and follow-up duration.

TableS8 Baseline characteristics of patients in IgM (-) and IgM-L cohort after matching

Variables	IgM (-)	IgM-L	*P*
N	266	266	
Male	97 (36)	107 (40)	0.42
Age, y	41 (31, 49.75)	39 (32, 49)	1
Systolic blood pressure, mmHg	119.5 (110.73, 131.17)	121.33 (112.58, 132.22)	0.12
Diastolic blood pressure, mmHg	74.33 (68.64, 80.2)	76 (69.01, 83)	0.33
Body mass index	22.6 (20.9, 24.57)	23.46 (20.81, 25.81)	0.09
Hemoglobin, g/L	126 (116, 138)	126 (115.12, 141)	0.76
Serum albumin, g/L	36.8 (33.73, 39.9)	36.7 (33.9, 39.85)	0.6
Serum creatinine, mmol/L	83 (65.25, 105.75)	81 (64, 109.75)	0.97
eGFR	89.31 (65.57, 111)	90.09 (63.43, 111.81)	0.86
Uric acid, mmol/L	349 (285.25, 418)	354 (308.55, 428.75)	0.23
Total cholesterol, mmol/L	4.9 (4.23, 5.64)	5 (4.33, 5.78)	0.31
Triglyceride, mmol/L	1.65 (1.16, 2.36)	1.62 (1.08, 2.21)	0.12
HDL-cholesterol, mmol/L	1.06 (0.86, 1.24)	1.11 (0.95, 1.32)	<0.01
LDL-cholesterol, mmol/L	2.75 (2.35, 3.34)	3 (2.54, 3.6)	<0.01
SII	6.05 (4.41, 8.35)	5.18 (3.95, 7.76)	<0.01
Serum IgA, g/L	3.29 (2.56, 4.27)	3.2 (2.55, 3.95)	0.12
Serum IgM, g/L	10.9 (8.75, 13.2)	10.1 (7.94, 12.28)	<0.01
Serum IgG, g/L	1.1 (0.74, 1.44)	1.06 (0.71, 1.47)	0.60
Serum C3, g/L	1.04 (0.91, 1.17)	1.06 (0.89, 1.21)	0.74
Serum C4, g/L	0.24 (0.19, 0.28)	0.23 (0.19, 0.28)	0.71
24h urinary protein, g/d	1.06 (0.52, 2.31)	0.95 (0.43, 1.82)	0.12
UACR, mg/g	343.65 (173.4, 1000.69)	324.94 (114.1, 905.83)	0.14
**Treatment**			
RAS blocker, n (%)	222 (83)	229 (86)	0.47
Glucocorticoid, n (%)	142 (53)	126 (47)	0.22
Immunosuppressant, n (%)	74 (28)	53 (20)	0.04
Cyclophosphamide	21 (7.9)	17 (6.4)	0.64
Mycophenolate Mofetil	28 (10.5)	17 (6.4)	0.13
Tripterygium Wilfordii	49 (18.4)	26 (9.8)	<0.01
**Pathology**			
M1	82 (44)	128 (60)	<0.01
E1	94 (51)	79 (37)	0.02
S1	130 (70)	138 (65)	0.32
T-score			0.04
1	57 (31)	42 (20)	
2	9 (5)	10 (5)	
C1	168 (64)	143 (55)	0.04
IgA			<0.01
1+	23 (9)	1 (0)	
2+	33 (12)	24 (9)	
3+	163 (61)	205 (77)	

4+	47 (18)	36 (14)	
IgM (1+), n (%)	0 (0)	266 (100)	<0.01
IgG			0.08
1+	6 (2)	16 (6)	
2+	9 (3)	13 (5)	
3+	6 (2)	3 (1)	
C3			<0.01
1+	23 (9)	1 (0)	
2+	33 (12)	24 (9)	
3+	210 (79)	241 (91)	
C4(1+)	1 (0)	0 (0)	1
**Follow up**			
Follow-up duration, m	27.9 (14.98, 45.63)	27.48 (15.79, 39.17)	0.83
TA SII	5.67 (4.11, 7.66)	5.13 (3.85, 6.68)	0.03
TA UACR, mg/g	306.56 (143.18, 758.88)	264.97 (106.07, 717.45)	0.21
Composite endpoint	16 (6)	7 (3)	0.09

Numerical variables are reported as mean ± SD or median (IQR), while categorical variables are presented as counts (%).

IgM-L represents IgAN patients whose IF intensity of IgM deposits was equal to 1+. IgM- refers to IgAN patients with the absence of IgM deposits. The composite endpoint was referred to a continuous decline in estimated eGFR from baseline of ≥50% or reaching ESRD.

E, endocapillary hypercellularity; M1, mesangial hypercellularity; T1-2; interstitial fibrosis/tubular atrophy; S, segmental glomerulosclerosis/adhesion; C1, crescent; UACR, urine albumin/creatinine ratio; SII, systemic immune-inflammation index; TA UACR, time averaged urine albumin/creatinine ratio; TA SII, time averaged immune-inflammation index.

P-value was adjusted for multiple comparisons.

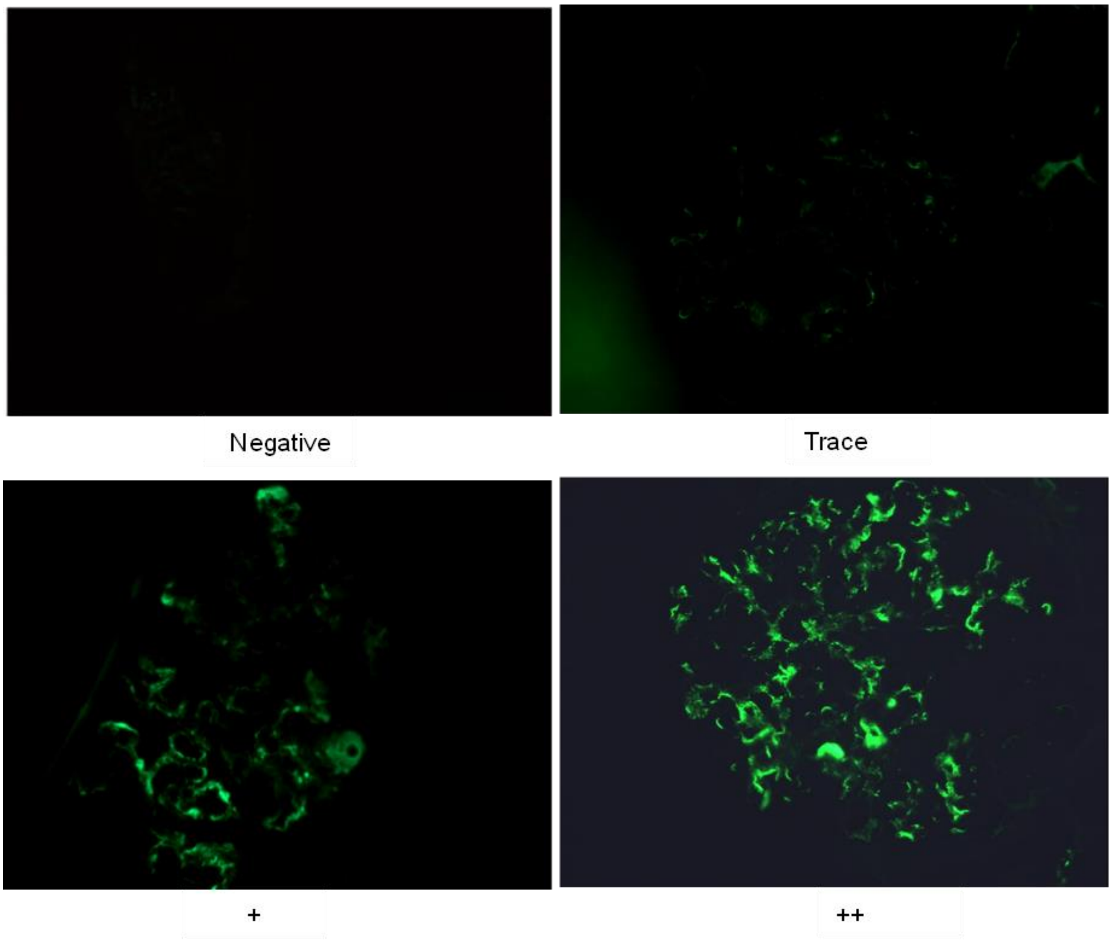

Figure S1 Renal biopsies with different intensities of IgM deposits in the mesangium

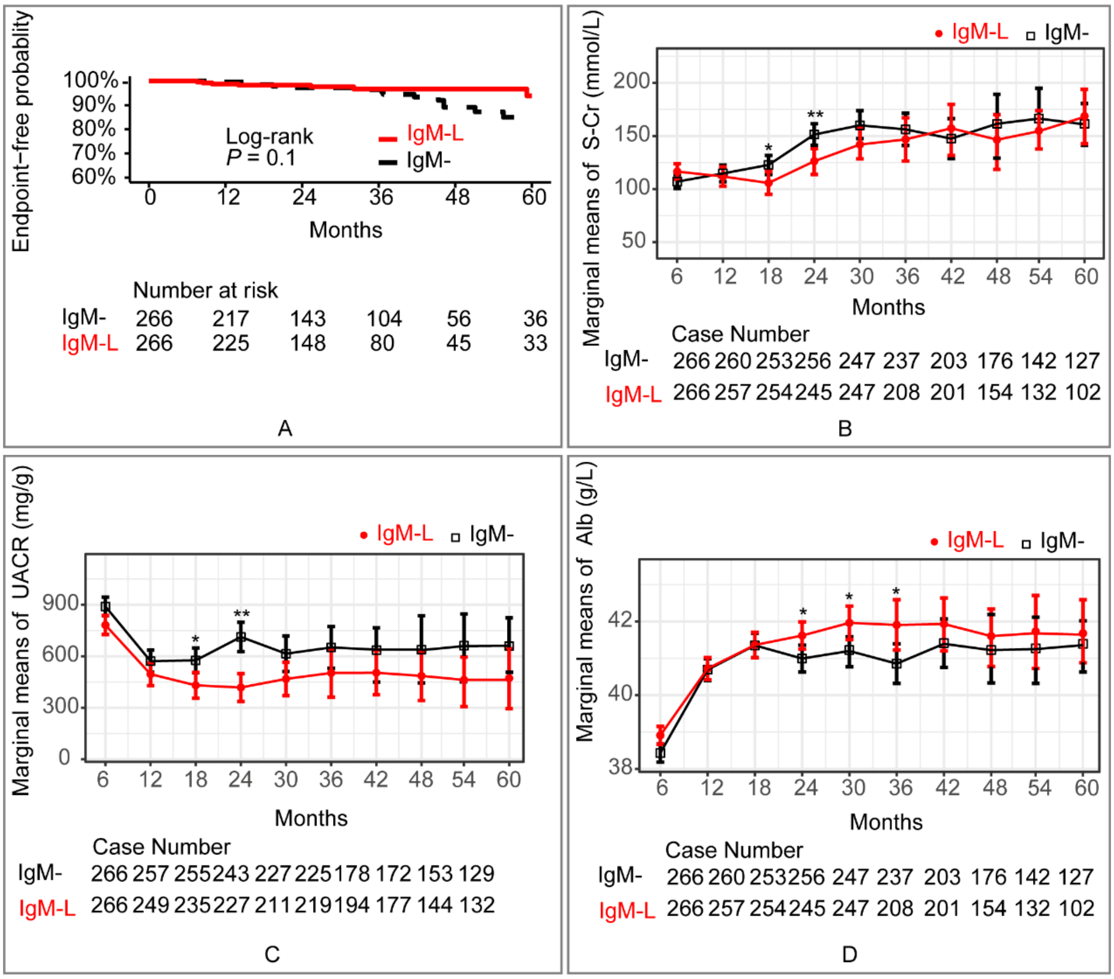

Figure S2 (A) Kaplan-Meier curve displayed the prognosis of the IgM-L and IgM- cohorts (*p*=0.1 [log-rank test]). The endpoint was defined as either a reduction of ≥50% in baseline eGFR or the development of ESRD. (B) Estimated marginal means and corresponding SEs of serum creatinine levels were estimated and compared between IgM-L and IgM- cohorts within the follow-up period using GLMM. (C) Estimated marginal means and corresponding SEs of urine albumin/creatinine ratio levels were estimated and compared between IgM-L and IgM- cohorts within the follow-up period using GLMM. (D) Estimated marginal means and corresponding SEs of serum albumin levels were estimated and compared between IgM-L and IgM- cohorts within the follow-up period using GLMM.

Statistical significance levels are indicated in the plot as '*', which represents $p< 0.05$, and '**', representing $p < 0.01$. The ANOVA results of the GLMM are presented in Table S7.

IgM-L represents IgAN patients whose IF intensity of IgM deposits was equal to 1+. IgM- refers to IgAN patients with the absence of IgM deposits. Alb, serum albumin; S-Cr, serum creatinine; UACR, urine albumin/creatinine ratio.
